# Early vaccine availability represents an important public health advance for the control of pandemic influenza

**DOI:** 10.1186/s13104-015-1157-1

**Published:** 2015-05-08

**Authors:** Amy L Greer

**Affiliations:** Department of Population Medicine, Ontario Veterinary College, University of Guelph, Guelph, ON N1G 2W1 Canada

**Keywords:** Influenza, Vaccination, Pandemic, Plant-based vaccines, Disease dynamics

## Abstract

**Background:**

Traditional processes for the production of pandemic influenza vaccines are not capable of producing a vaccine that could be deployed sooner than 5–6 months after strain identification. Plant-based vaccine technologies are of public health interest because they represent an opportunity to begin vaccinating earlier.

**Methods:**

We used an age- and risk- structured disease transmission model for Canada to evaluate the potential impact of a plant-produced vaccine available for rapid deployment (within 1–3 months) compared to an egg-based vaccine timeline.

**Results:**

We found that in the case of a mildly transmissible virus (*R0* = 1.3), depending on the amount of plant-based vaccine produced per week, severe clinical outcomes could be decreased by 60–100 % if vaccine was available within 3 months of strain identification. However, in the case of a highly transmissible virus (*R0* = 2.0), a delay of 3 months does not change clinical outcomes regardless of the level of weekly vaccine availability. If transmissibility is high, the only strategy that can impact clinical outcomes occurs if vaccine production is high and available within 2 months.

**Conclusions:**

Pandemic influenza vaccines produced by plants, change the timeline of pandemic vaccine availability in a way that could significantly mitigate the impact of the next influenza pandemic.

## Background

The 2009 (H1N1) influenza pandemic represented a unique public health challenge for the world. In most people, the H1N1 pandemic strain caused relatively mild, self-limiting disease while others experienced severe disease requiring hospital and/or ICU admission, prolonged ventilation and supportive care, and some individuals died as a result of their infection [1-3]. Governments, healthcare providers and facilities, along with industry had been preparing best practices and strategies for mitigating the potential impact of a pandemic for many years prior to the emergence of the pandemic H1N1 virus in Mexico and subsequent global spread [4-6]. Globally, countries have developed pandemic plans that include descriptions of available mitigation strategies and plans for deploying available public health resources and interventions such as vaccines, and antivirals, along with community interventions such as school closures and other types of social distancing [7]. Vaccination has always formed the cornerstone of public health interventions and pandemic influenza is no different. In this case, the development of a pandemic influenza vaccine begins when the viral strain has been fully sequenced and the strain information released to vaccine manufacturers. Once a suitable vaccine candidate has been developed, regulatory processes are in place to verify that the vaccine candidate is both safe and effective. Once the vaccine has been approved for use, public vaccination campaigns can begin using a vaccine prioritization strategy based on the epidemiology of the disease in the population.

Most vaccine manufacturers who develop influenza vaccines use 9–12 day old embryonated eggs to produce the vaccine. The vaccine strain is injected into the eggs where it replicates over several days while the eggs are incubated. After incubation, the egg contains many millions of vaccine virus particles that are purified to produce the antigen that will be further processed to produce the vaccine [8]. The limiting step in this process is the number of eggs that each manufacturer can procure, inoculate and incubate at a time. Another issue is that some strains of influenza virus do not replicate well in eggs and as a result, it is difficult to produce enough antigen to manufacture large amounts of vaccine for distribution. As demonstrated in 2009, the full process of vaccine production, safety testing, clinical trials and regulatory approval optimistically takes between 5-6 months (and could be longer for strains that grow more poorly in eggs). Recent advances in virology and immunology have resulted in many novel techniques that could revolutionize the way we produce vaccines. One such strategy focuses on the expression of recombinant protein in the cells of tobacco plants [8, 9]. This plant-based production system produces yields consistent with an industrial process and these yields are achieved in a relatively short period of time (suggested to be within 3 weeks of the release of the pandemic strain) [10, 11]. Wirz et al. [12] describe a plant-based vaccine production system that is able to produce virus-like particles within weeks of production start-up using an automated production system. More recently, in response to the circulation of a highly virulent strain of influenza virus (H7N9) in China which was first reported in March 2013, Medicago Inc. (a clinical stage, biopharmaceutical company) was able to produce the first lot of plant-produced vaccine for pre-clinical trials only 19 days after the sequence was first accessed [13]. Importantly, data emerging from phase 1 clinical trials of plant-produced influenza vaccines appear to suggest that these vaccines are highly immunogenic and safe [10, 14, 15]. Due to the expected shortened time frame for vaccine production, plant-made, virus-like particle influenza vaccines represent an important opportunity for protecting public health in the case of the next influenza pandemic.

For this study, we have evaluated the proposed vaccine timing and expected range of plant-based vaccine production capabilities on pandemic influenza outcomes using a mathematical model. Specifically, we used a dynamic, age- and risk- stratified SEIR model for the transmission of pandemic influenza within the Canadian population. This approach allows us to examine the interactions that exist between the time at which a pandemic influenza vaccine is available to the public, and the transmission characteristics of the pandemic strain. We will examine how these interactions affect the projected clinical outcomes observed in the simulations such as changes in the clinical attack rate, hospitalizations, and deaths using a mathematical model.

## Methods

### Dynamic model

The model was developed using the dynamic simulation tool, AnyLogic **©** (V.7.0) (St. Petersburg, Russia). The base model is a system dynamic, Susceptible (*S*) – Exposed (*E*) – Infected (*I*) – Recovered (*R*) model similar to a previously published model describing the transmission of pandemic influenza within Canada [16]. The infectious (*I*) compartment is broken down into two different components. The first represents individuals who are infectious but asymptomatic (*I*_*A*_) and the second represents individuals who are infectious and symptomatic (*I*_*S*_). We assumed that 40 % of all cases were asymptomatic [17] and that all infected individuals were equally infectious. In addition, two outcome compartments are included. These are Hospitalized (*H*) and Death (*D*). This permits us to keep track of two specific clinical outcomes of interest. Symptomatically infectious individuals (*I*_*s*_) are able to recover from their infection or alternatively, may require hospitalization, in which case they transition to the (*H*) compartment. From the Hospitalized (*H*) compartment, individuals may move to the recovered compartment (*R*) or may die as a result of their infection and move to the Death compartment (*D*). We assume that deaths only occur in patients who have severe enough disease to require hospitalization. The model runs for a time period of 12 months and therefore we did not consider the immigration or emigration of individuals, nor did we consider population aging.

### Model structure (age and risk groups)

The model population is broken down into seven different age classes with the following cut-offs: 0–4 years, 5–13 years, 14–17 years, 18–22 years, 23–52 years, 53–64 years and ≥65 years. Data on the population demographics was obtained from Statistics Canada [18]. During the 2009 pandemic, older individuals appeared to have some level of pre-existing immunity due to exposure to an antigenically similar virus earlier in their life [19]. Since it is not possible to know if this may be the case in a future pandemic we chose to only examine scenarios where the level of pre-existing immunity was 0 % (Table 1) in order to consider a “worst-case” scenario. Mixing between the different age groups was parameterized based on data collected by Mossong et al. [20]. Each of the age classes is further subdivided into two health states. Individuals are classified as either healthy or as having one or more underlying chronic medical conditions for which seasonal influenza vaccine is recommended. The chronic conditions considered in the model were based on data from the Canadian Community Health Survey [21]. State transitions were the same for healthy individuals and individuals with chronic conditions however, individuals with chronic conditions had an enhanced risk of having a poor clinical outcome (e.g. hospitalization or death as a result of their infection). During the 2009 pandemic pregnant women were also considered to be at high risk for complications from pandemic influenza [3]. As a result, we have included a separate pregnancy state that represents women in the second or third trimester of pregnancy. Estimates of the number of pregnant women at any point in time were calculated using data from Statistics Canada on pregnancies and live births [22].

**Table 1:** Vaccination scenarios to be examined using the dynamic model

	Scenarios 1 and 2	Scenarios 3 and 4	Scenarios 5 and 6
Pandemic transmissibility*	Mild/Severe	Mild/Severe	Mild/Severe
Season of emergence	Spring/Fall	Spring/Fall	Spring/Fall
Level of pre-existing immunity in level of pre-existing immunity in population	0%	0%	0%
Time at which first vaccine becomes available (range)	6 months	1 month (1–3 months)	1 month (1–3 months)
Time at which additional vaccine becomes available	_	_	6 months
Doses of traditional (egg-based) vaccine required	1/2	_	1/2
Doses of novel (plant-based) vaccine required	_	1/2	1/2
Vaccine coverage*	UIIP	UIIP	UIIP
Vaccine production for traditional vaccine	3.75 M doses/week		3.75 M doses/week
Vaccine production for novel vaccine		150,000 doses/week1.5 M doses/week	150,000 doses/week1.5 M doses/week
Outcomes**	CPIP moderate	CPIP moderate	CPIP moderate

*See Table 2 for details regarding specific parameter values for pandemic influenza transmissibility scenarios and vaccine coverage.

**See Table 2 for details.

### Seasonal effects

Influenza demonstrates marked seasonality with disease occurrence primarily in winter months [23, 24]. This seasonality influences the expected wave pattern of pandemic influenza. To model the seasonal dynamics of a potential influenza pandemic we include reduced transmission over the course of the summer, when typical influenza seasonality and changes in contact patterns may reduce the basic reproductive number (R0). The base model for a spring emergence starts with the Canadian pandemic strain introduced to Canada in April (similar to the 2009 pandemic). This generates the characteristic, 2-wave pattern seen in past pandemics (a small, herald wave in the spring followed by a larger fall wave). For comparison, the model is adjusted to look at the impact of a pandemic influenza strain introduction that occurs in the fall (resulting in a single large wave of influenza cases).

### Vaccination strategies

Vaccination in the model assumes a risk/outcome based vaccination strategy similar to the vaccine prioritization observed in Canada during the 2009 pandemic. Vaccine prioritization in the model is therefore as follows: 1) pregnant women and all individuals with a chronic underlying condition as defined by the CCHS (regardless of age), 2) healthy children aged 0–4 and healthy adults aged 65+, 3) healthy children aged 5-17, and 4) healthy adults aged 18–64.

We assume that pandemic vaccine uptake would be similar to seasonal influenza vaccine uptake in Ontario, where influenza vaccine is available free of charge through the universal influenza immunization program (UIIP) to any individual wishing to receive the vaccine who is 6 months of age or older (Table 1) [25, 26]. We have considered two different dosing schedules with individuals receiving one dose or two doses of pandemic influenza vaccine. In the case of a severe pandemic (virus is easily transmissible and/or clinically severe), we assume that individuals would require two doses whereas if the pandemic strain were less severe (only mildly transmissible and/or causing mild to moderate clinical symptoms) we assume that individuals could be vaccinated with only one dose. For scenarios using a one-dose schedule, we assume that full immunity occurs 10 days after vaccination. In comparison, for scenarios using a two-dose schedule, we assume that full immunity occurs immediately after vaccination with the second dose. We do not consider the effect of partial protection following the first dose in the two-dose schedule.

The fraction of the population that acquires immunity is based on vaccine effectiveness (VE) estimates for two different age groups (those older than 65 years and those less than 65 years). We assume that the effectiveness of the pandemic vaccine is similar to that of seasonal influenza vaccine with lower effectiveness in older individuals (Table 1).

Novel, clinical-stage vaccine candidates produced using plants may be available for use in the case of the next influenza pandemic. We expect that there will be different types of pandemic vaccine available on the Canadian market including both traditionally produced influenza vaccine (egg-based), and novel, plant-produced vaccines. It is expected that the time from the availability of the pandemic strain to manufacturers until a pandemic vaccine is available in the community is significantly shorter for a vaccine that is produced using a plant-based technology rather than an egg-based technology. Egg-based vaccine technology takes approximately 6 months from strain identification to having vaccine vials available for public use [16] whereas, we assume that a plant-based vaccine could likely be produced in 1–3 months. Based on publically available data for traditional, egg-based pandemic vaccine production by GlaxoSmithKline (GSK) in Canada, we assume that 3.75 million doses of pandemic vaccine would be available per week once the vaccine was approved for use (Health Canada, 2013). For comparison, we have evaluated both a low production scenario and a high production scenario for a plant-produced vaccine. In the base case, we assume that a plant-produced vaccine enters the Canadian market at 150,000 doses/week and that the upper bound for production capability is 1.5 million doses/week. For all vaccine scenarios, we do not consider the logistical time lags associated with distributing vaccine across Canada.

## Results

A total of 225 simulations were run using the dynamic model representing different combinations of vaccination strategies (vaccine coverage, type of vaccine, timing of vaccine availability, and vaccine production levels) and different pandemic scenarios (season of emergence, and transmissibility) (Table 1). The model outputs included epidemic curves for each scenario and the clinical attack rate for the first 12 months of the simulated pandemic. Using the model outputs we also projected the number of possible hospitalizations and deaths for each scenario based on assumptions published in the Canadian Pandemic Influenza Plan (CPIP) regarding the potential impact of a pandemic of moderate clinical severity [7].

**Fig. 1:**
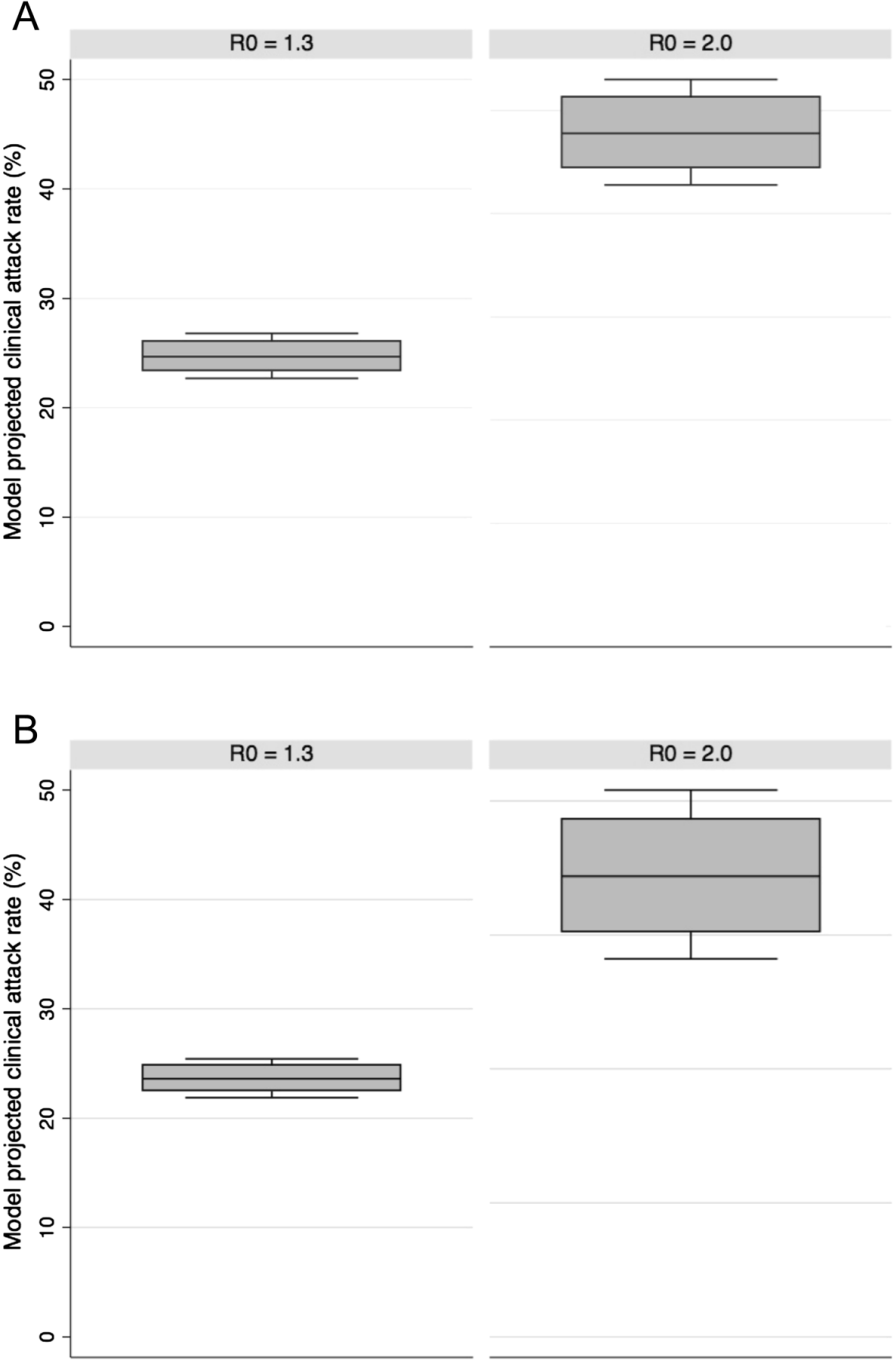
The model projected clinical attack rates (%) for all combinations of simulation runs for a simulated Canadian pandemic that emerges in fall (top - **A**) or spring (bottom - **B**) for two different transmissibility values (1.3 and 2.0)

In general, simulation results for scenarios in the absence of any pandemic interventions were in line with previously published work describing influenza transmission in a mild pandemic (e.g. 2009) and a severe pandemic (e.g. 1918) [27-32]. Simulations with an R0 of 1.3 resulted in lower clinical attack rates (<30 %) than those observed in simulated pandemics with an R0 of 2.0 (<50 %) (Fig. 1). In addition, scenarios where the pandemic strain emerges in Canada in the spring (Fig. 1B) exhibit lower overall clinical attack rates than scenarios where the pandemic emerges in the fall (Fig. 1A).

### The impact of traditional pandemic vaccine

**Fig. 2:**
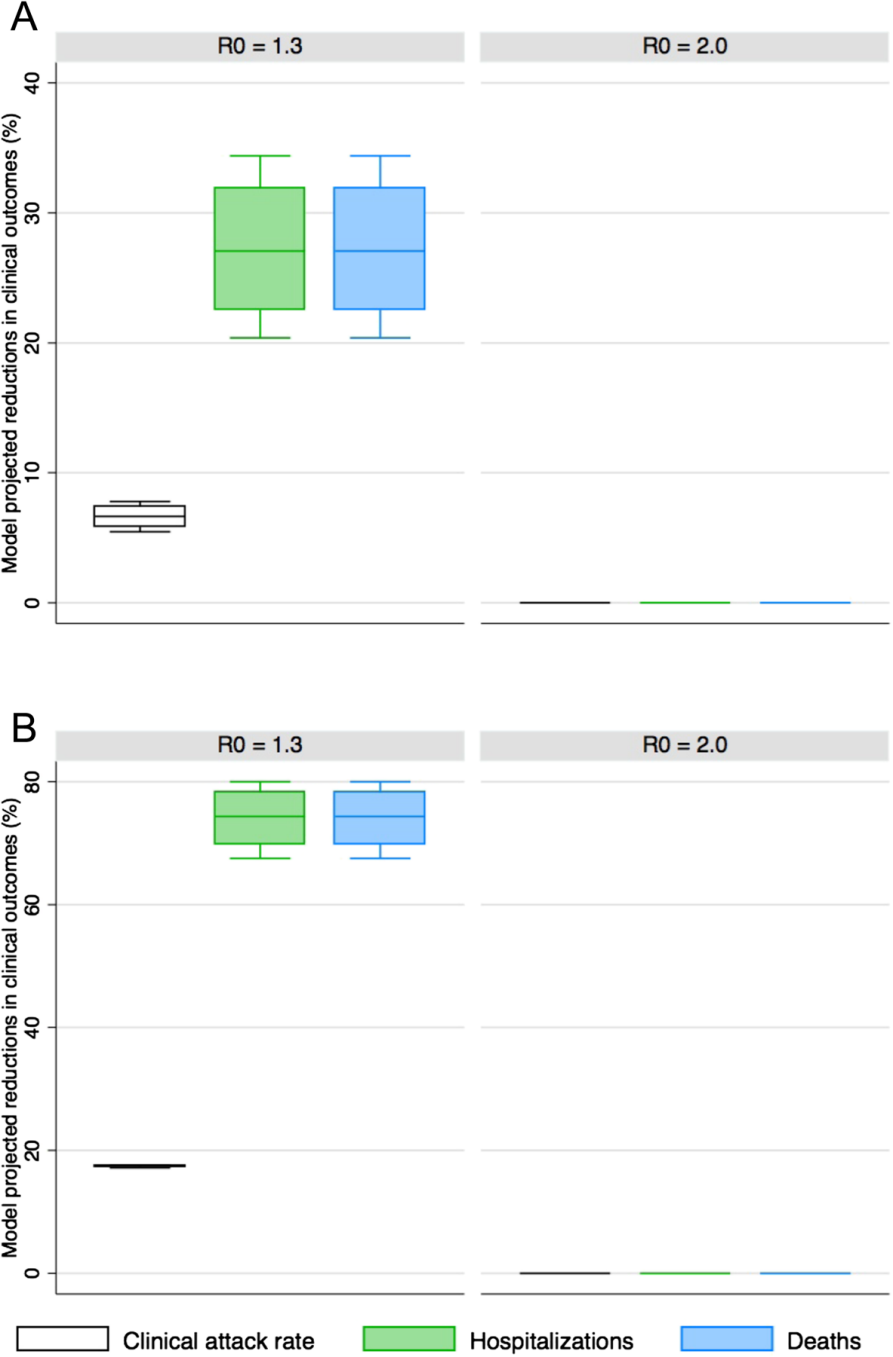
The percent reduction in clinical outcomes for a scenario where traditional pandemic influenza vaccine begins to be deployed 6 months after the emergence of the pandemic strain in Canada relative to a scenario with no vaccine available (base case = no interventions). Results are broken down by season of emergence (**A** = fall, **B** = spring)

For scenarios where the only vaccination option is traditional pandemic influenza vaccine with availability starting 6 months after the emergence of the pandemic strain and 3.75 million doses available each week, the ability of vaccination to reduce adverse clinical outcomes (clinical attack rate, hospitalizations and/or deaths) is poor if the pandemic is highly transmissible (*R0* = 2.0) regardless of the season of emergence (Fig. 2). In this case, such a significant proportion of the population has already been infected by the time vaccine becomes available that the percent reduction in clinical outcomes compared to the baseline scenario (with no interventions) is essentially zero.

For a mild pandemic (*R0* = 1.3), vaccination beginning at 6 months is able to reduce clinical outcomes such as hospitalization and deaths primarily by prioritizing the vaccination of high-risk individuals. This can be seen in Fig. 2, where reductions in projected hospitalizations and deaths range from approximately 20–80 % depending on the season of pandemic emergence. However, it is important to point out that since vaccine only becomes available at 6 months and vaccination is prioritized to high-risk individuals, vaccination beginning at the 6 month time point is not sufficient to reduce the overall clinical attack rate (Fig. 2).

### The potential impact of a novel plant-based pandemic vaccine

**Fig. 3:**
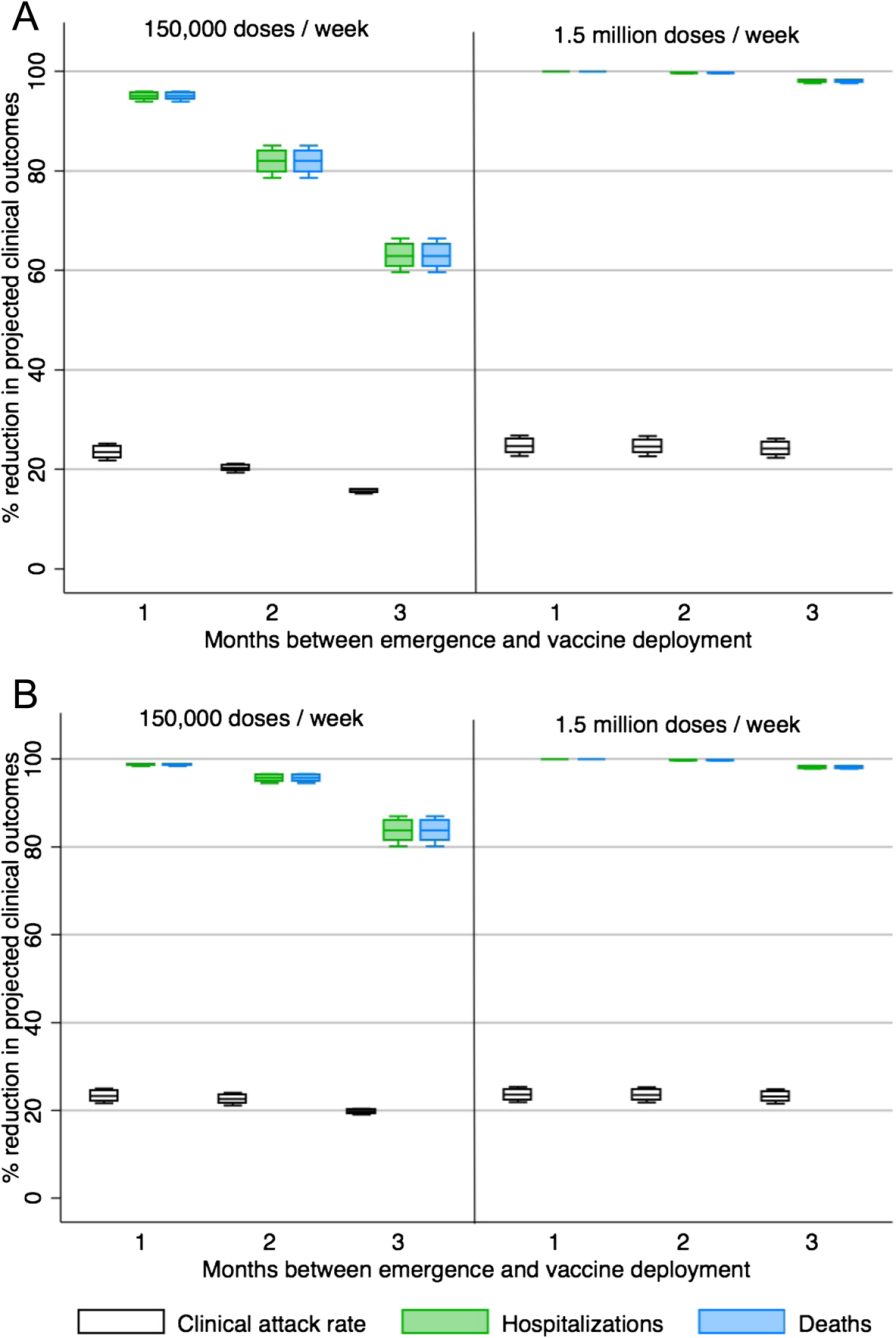
The percent reduction in clinical outcomes for a scenario where a plant-produced pandemic influenza vaccine could be deployed 1, 2 or 3 months after the emergence of the pandemic strain (*R0* = 1.3) in Canada relative to a scenario with no vaccine available. Results are broken down by season of emergence (**A** = fall, **B** = spring)

**Fig. 4:**
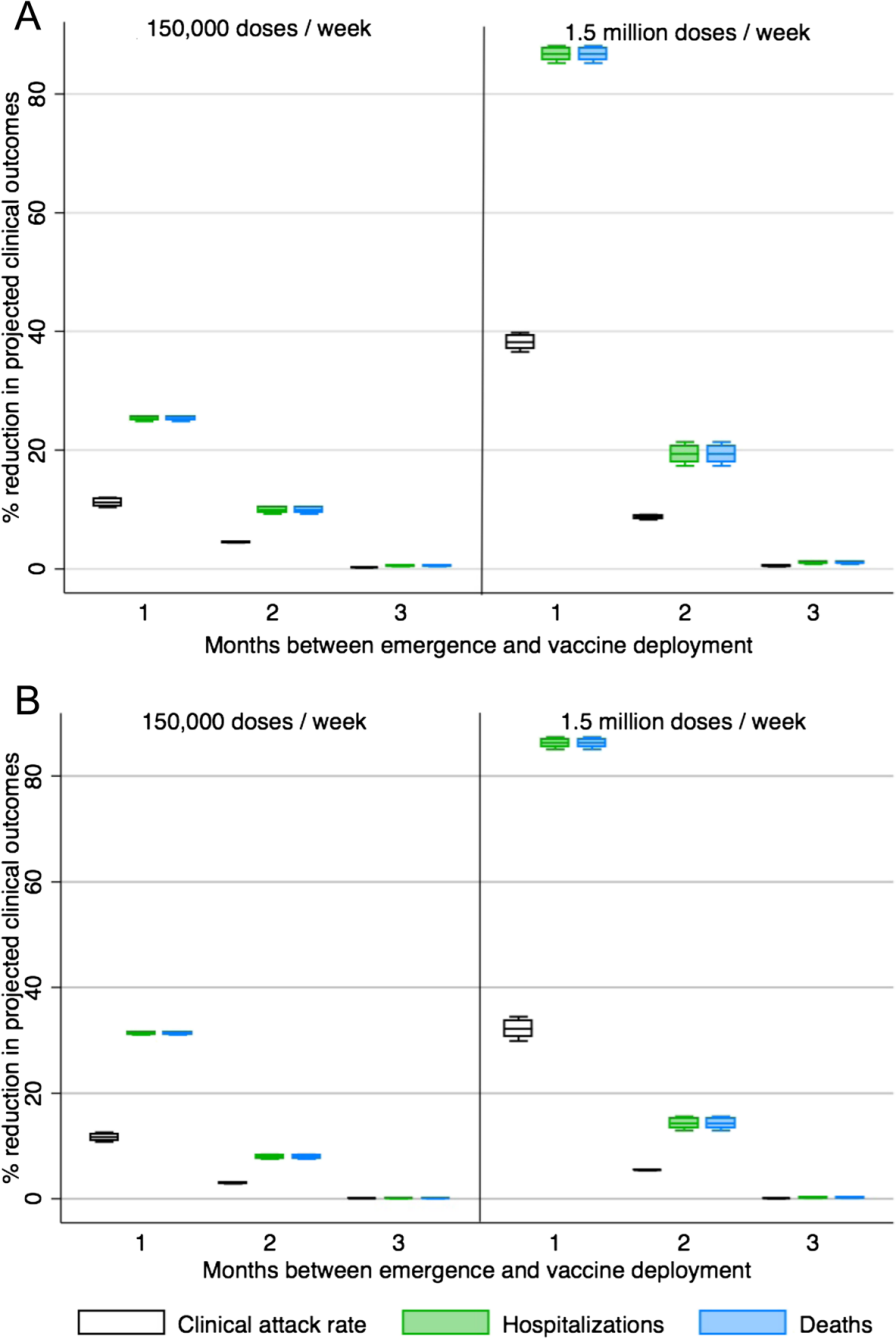
The percent reduction in clinical outcomes for a scenario where plant-produced pandemic influenza vaccine begins to be deployed 1, 2 or 3 months after the emergence of the pandemic strain (*R0* = 2.0) in Canada relative to a scenario with no vaccine available. Results are broken down by season of emergence (**A** = fall, **B** = spring)

In a situation where a safe and effective pandemic vaccine could be produced and become available earlier in a pandemic than traditional pandemic vaccine a significant impact can be seen in the ability of a vaccination program to reduce serious adverse clinical outcomes. This is the case for both a mild pandemic and more severe pandemic (Figs. 3 and 4). More moderate reductions can be seen in the reduction of overall clinical attack rates (Figs. 3 and 4).

In the case of a mild pandemic (*R0* = 1.3) where plant-based vaccine production is 150,000 doses/week, if vaccine deployment begins between 1 month and 3 months post emergence (3–5 months earlier than current traditional pandemic vaccine availability), hospitalizations and deaths could be reduced 60–100 % compared to the base case over the course of a year long pandemic regardless of season of emergence (Fig. 3). If plant-based vaccine production were higher (1.5 million doses/week), hospitalizations and deaths could be almost entirely avoided if vaccine became available within 3 months of emergence in Canada. Transmission would still occur within the population as seen by a relatively steady clinical attack rate (Fig. 3), however, because vaccine would be available to vaccinate the most high risk individuals early in the pandemic, serious morbidity and mortality could be avoided.

If the transmissibility of the pandemic strain was relatively high (*R0* = 2.0), earlier vaccine availability is of key importance for reducing population morbidity and mortality. In this case, a delay of 3 months will have only a very minimal impact on clinical outcomes (Fig. 4) regardless of the vaccine production levels examined in the model. However, if vaccine production levels are high (1.5 million doses/week) and vaccine became available between 1 and 2 months post emergence, a vaccination program could reduce clinical outcomes significantly (Fig. 4). In this case, the differences observed between a fall emergence (Fig. 4A) and a spring emergence (Fig. 4B) are minimal.

### The impact of plant-based vaccines combined with traditional vaccines

For almost all scenarios, the impact of the 2 different vaccines together is similar to scenarios 3 & 4 (Table 1) where only plant-based vaccine is available. This is because, if plant-based vaccine is available early (within 1–2 months of viral emergence) and production levels are high, the vaccine coverage levels examined here (Table 2) are met before traditional vaccine becomes available at 6 months. For scenarios where plant-based vaccine becomes available at 3 months or the plant-based vaccine production levels are low, there are several weeks of overlap between the availability of the two different vaccines (plant-based and traditional) in order to achieve the population vaccine coverage levels examined in the model.

**Table 2:** Model parameter values

Variable	Age group(s)	Value	Source
Total population size	All	33,739,859	[47]
Proportion of the population with pre-existing immunity	53 years +	0%	[48]
**Mild pandemic scenario**			
Latent period (days)	All	3.5 days	[16, 27]
Duration of Infectiousness (days)	All	2.5 days	
Reproductive Number	All	1.3	
**Severe Pandemic Scenario**			
Latent period (days)	All	1.9 days	[28, 49]
Duration of infectiousness (days)	All	4.1 days	[28, 49]
Reproductive number	All	2.0	[28]
**Vaccine scenarios**			
Vaccine effectiveness (traditional influenza vaccine)	<65 years	0.7	[50]
	≥65 years	0.3	
Vaccine effectiveness (plant-based pandemic influenza vaccine)	<65 years	0.7	Assumption
	≥65 years	0.3	
Proportion of the population with high-risk conditions	0–4 years	0.10	[21, 25]
	5–13 years	0.10	
	14–17 years	0.12	
	18–22 years	0.11	
	23–52 years	0.13	
	53–64 years	0.27	
	≥65 years	0.43	
Proportion of the population vaccinated	0–4 years	0.26	[25, 26]
	5–13 years	0.30	
	14–17 years	0.31	
	18–22 years	0.29	
	23–52 years	0.29	
	52–64 years	0.47	
	≥65 years	0.75	
Percentage of clinically ill individuals who are hospitalized and recover	All	1%	[7]
Hospitalized cases that die	All	0.4%	[7]

**Fig. 5:**
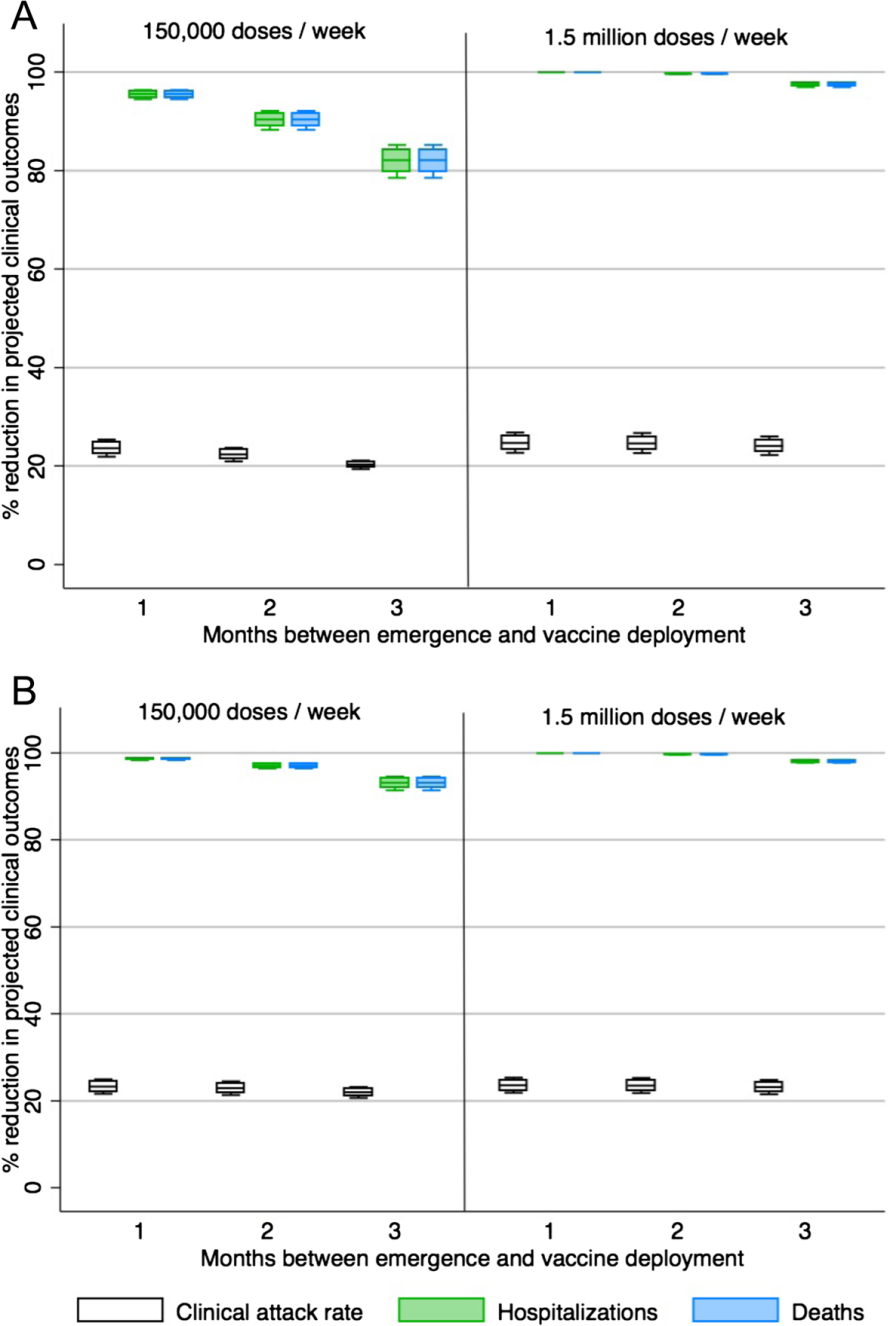
The percent reduction in clinical outcomes for a scenario where plant-based pandemic influenza vaccine begins to be deployed 1, 2 or 3 months after the emergence of the pandemic strain (*R0* = 1.3) in Canada combined with the availability of traditional pandemic vaccine beginning at 6 months relative to a scenario with no vaccine available. Results are broken down by season of emergence (**A** = fall, **B** = spring), and expected vaccine production levels (for plant-based vaccines)

**Fig. 6:**
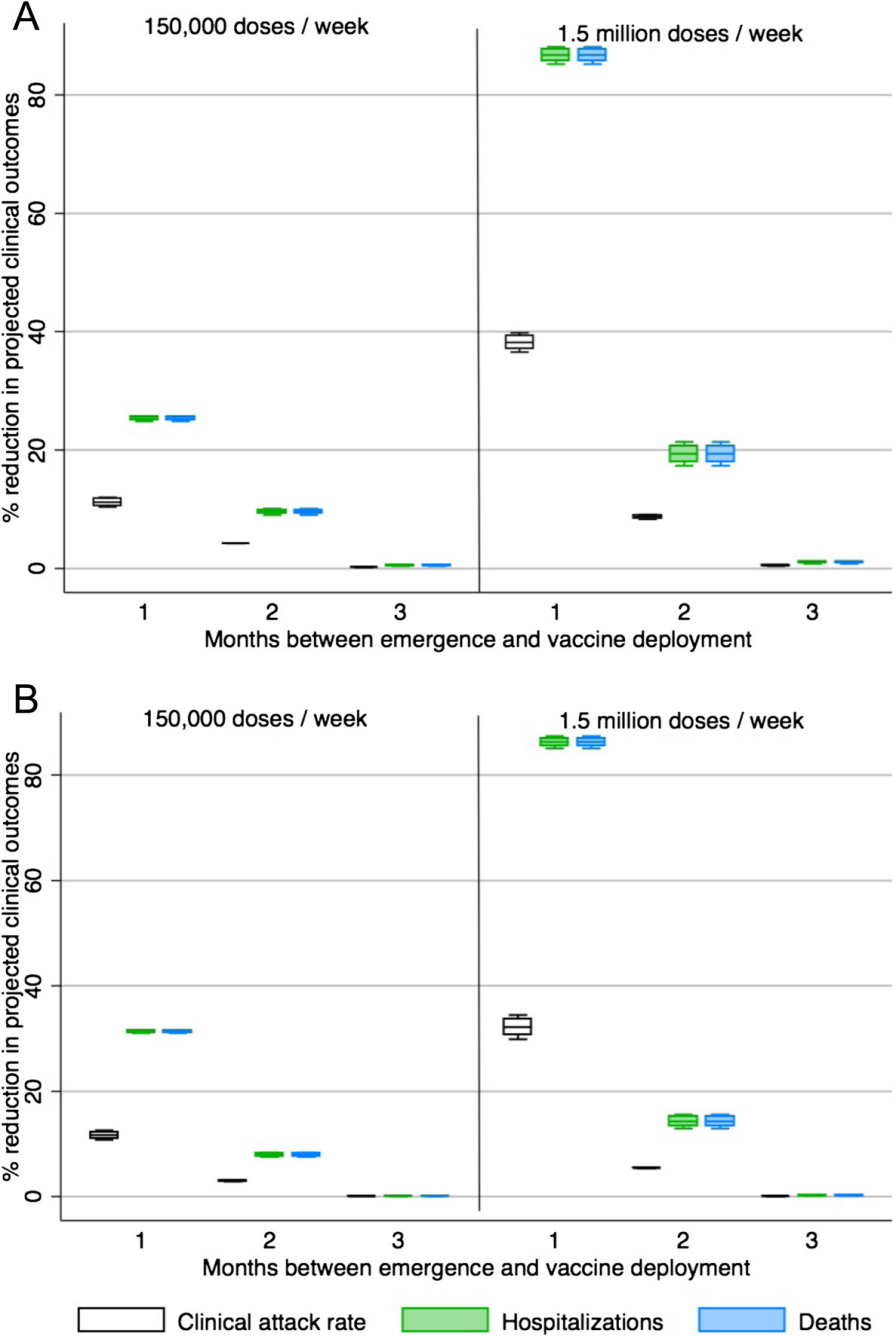
The percent reduction in clinical outcomes for a scenario where plant-produced pandemic influenza vaccine is deployed 1, 2 or 3 months after the emergence of the pandemic strain (*R0* = 2.0) in Canada combined with the availability of traditional pandemic vaccine at 6 months relative to a scenario with no vaccine available. Results are broken down by season of emergence (**A** = fall, **B** = spring), and expected vaccine production levels (for plant-based vaccines)

In these simulations, projected hospitalizations and deaths could be reduced more than 80 % even if the production of plant-based vaccines is at low levels (150,000 doses/week), if vaccine is available early and the pandemic is relatively mild (Fig. 5). If, plant-based vaccine production was 1.5 million doses per week and vaccine was available early, adverse outcomes could almost entirely be avoided if the pandemic was mild (Fig. 5). Incorporating both plant-based vaccine (with low production levels per week) and traditional vaccine (at 6 months) has a more significant impact on reducing hospitalizations and deaths than plant-based vaccine alone if the production of plant-based vaccines is at low levels (Figs. 3 and 5). In the case of a severe pandemic (*R0* = 2.0), the impact of adding traditional vaccine at 6 months time does not change the overall impact of vaccination on hospitalizations, deaths, or clinical attack rates from scenarios where only plant-based vaccine is available (Figs. 4 and 6).

## Discussion

Decreasing the time between pandemic influenza virus emergence and the time at which a safe and effective vaccine is available for distribution can have a significant impact on public health [16, 33-35]. The 2009 (H1N1) influenza pandemic highlighted the fact that producing large amounts of vaccine in a short amount of time using existing egg-based technologies is difficult. This model demonstrates that early vaccine availability could significantly decrease pandemic influenza morbidity and mortality by more than 50 % in the case of a mild pandemic with low, plant-based vaccine production capabilities (150,000 doses/week) and up to 100 % if plant-based production capacity was high (~1.5 million doses/week). In the case of a severe pandemic, reductions in pandemic influenza morbidity and mortality between 15 and 60 % may be possible with low, plant-based vaccine production capabilities and up to 90 % if production capabilities were high. If the pandemic strain was highly transmissible (*R0* = 2.0), and no other intervention strategies were available, early vaccine availability is the only way to reduce clinical outcomes such as hospitalization and death. Late vaccine availability in a scenario where the pandemic strain is highly transmissible is not able to reduce pandemic associated hospitalizations or deaths.

Shortening the time between viral emergence and the start of a national vaccine program has a more significant impact on averting population morbidity and mortality than on population clinical attack rates. We based our simulated vaccine programs on a prioritization plan similar to that seen in Canada during the 2009 pandemic [16]. The vaccine program would prioritize individuals with underlying chronic health conditions followed by individuals at increased risk of suffering an adverse influenza associated outcome followed by all other healthy individuals [16]. Alternative prioritization strategies have not been evaluated in the current simulations but may be worth considering especially given the added benefit that may be possible if plant-produced vaccine could be produced and distributed rapidly and in large quantities early in a pandemic.

Previous modeling work has demonstrated that if a pandemic vaccine were available early in a pandemic, prioritizing influenza “transmitters” such as young, healthy children rather than individuals at highest risk of suffering a severe outcome such as hospitalization or death could provide more benefit than the typical influenza prioritization plan which focuses on individuals at highest risk of suffering an adverse outcome [36]. These alternative vaccine prioritization strategies should be further evaluated in the context of both the timing and production capabilities of emerging plant-based vaccine production technologies.

We have used conservative estimates of vaccine effectiveness in our simulations. We have set vaccine effectiveness values to be the same for traditional vaccine and the plant-based vaccine in order to focus our results on the projected impact of differential vaccine timing. However, if the vaccine effectiveness estimates for the plant-based vaccine were higher than those examined here, especially for older individuals for whom traditional influenza vaccines are only weakly immunogenic [37], the reduction in clinical outcomes compared to the base case could be even greater than what we have described. Preliminary clinical data suggest that influenza vaccines containing plant-made, virus-like particles may demonstrate improved vaccine effectiveness [10, 38, 39]. As more research regarding the outcomes of human clinical trials of plant-made vaccines containing virus-like particles (VLP) becomes available, realistic vaccine effectiveness estimates for these novel vaccines can be incorporated into the existing mathematical models for pandemic influenza. We would expect that if these novel vaccines were able to elicit an improved immune response, especially in individuals older than 65 years of age, the results of this modeling exercise could be considered conservative estimates.

We have also used conservative estimates for the upper bound of plant-based vaccine availability. In our simulations, we assumed that the maximum production capability would be 1.5 million doses available per week for a plant-based vaccine. Our results demonstrate that at this level of production, significant protection can be provided to the population especially when vaccine is available early. Recently, an industrial virus-like particle, plant-based vaccine production facility built in Research Park Triangle, NC has demonstrated that it was capable of producing 10 million doses of an influenza vaccine candidate in 1 month (2.5 million doses per week). The US Defense Advanced Research Projects Agency (DARPA) funded this production challenge test as an initiative to encourage industry to develop scalable processes that would enable the rapid production of immunogenic vaccines for emerging and novel biological threats [40].

As with all mathematical models, this model includes simplifying assumptions and incorporates parameter values that are subject to some uncertainty. The model presented examines a range of pre-defined scenarios and does not have the ability to identify the likelihood that a given scenario will or will not occur. All of the scenarios examined here base the vaccine timing on the virus emerging in Canada. It is likely that if the pandemic strain were to emerge outside of Canada (e.g. southeast Asia) vaccine development would begin before the virus was imported to Canada. If this were the case, it may permit earlier vaccine availability even using traditional, egg-based vaccines. However, research has demonstrated that given the highly connected nature of the global population, regardless of where the pandemic originates, global spread is likely to occur rapidly [41-43]. This highlights the need for a pandemic vaccine production system that is flexible and can respond quickly in the case of a pandemic such that public health vaccination programs could begin earlier than the typical 5–6 months after the emergence of the pandemic strain.

We have examined two different scenarios related to the emergence of a pandemic influenza virus in Canada (spring and fall). The timing of the peak of the pandemic wave in relation to vaccine availability for a national vaccine campaign is a critical factor. We have examined these two scenarios as examples to illustrate the potential impact of vaccine timing however, changes to peak timing will impact the outcome of a vaccination program either positively or negatively.

Our vaccination simulations did not specifically address the possibility of providing vaccination for individuals working in critical infrastructure such as healthcare or others who would need to remain on the job during a pandemic. We assume that all individuals in the population would be vaccinated based on the described prioritization list and coverage levels (Table 1). We feel that this is a reasonable approximation of population vaccine coverage. Even if vaccination were recommended for specific sectors, individuals who would typically not accept seasonal influenza vaccine are unlikely to accept a pandemic vaccine unless the pandemic were especially severe. We also know that mandating influenza vaccine for specific sectors such as healthcare workers has met with significant resistance from employees and employee unions but in some circumstances may be a useful strategies to consider [44-46]. Our model does not specifically address other outcomes associated with vaccine programs such as societal disruption and/or economic costs. It is also important to recognize that even though we have assumed that a plant-produced vaccine could be available between 1 and 3 months after the pandemic virus emerges; we have not considered the potential regulatory delays associated with vaccine approval, which would impact the actual release of the vaccine to the public.

We did not consider the effect of spatial heterogeneity or the time required to distribute vaccine across the country, which could result in delays in vaccine program start times not considered by this model. We also did not incorporate other concurrent mitigation strategies on influenza transmission, including antivirals and social distancing measures. We did not consider the impact of co-circulating seasonal influenza strains. To address the uncertainty in our estimates of mortality and hospitalization rates, due to both the low frequency of occurrence of these outcomes and reporting biases and other limitations inherent in surveillance data, we have focused our analysis on qualitative results.

## Conclusions

Using a mathematical model that simulates the transmission of pandemic influenza virus within Canada, we have demonstrated that vaccine timing is of critical importance. Given the highly connected nature of the global population, it is unlikely that countries will have significant lead-time for the development and distribution of a pandemic vaccine before clinical cases are identified. Regardless of where the next pandemic virus emerges, the ability to rapidly develop and provide access to a safe and effective pandemic influenza vaccine is of importance for the control of pandemic influenza. Vaccine remains the primary mechanism to prevent serious clinical outcomes in the most vulnerable groups. Our results demonstrate that early public access to pandemic influenza vaccine within the first several months of an influenza pandemic, as opposed to the traditional timeline currently assumed using traditional egg-based influenza vaccine production platforms has the ability to prevent a significant number of severe clinical outcomes such as hospitalization and death. Pandemic vaccine production platforms that utilize plant-based technologies represent an important opportunity for meeting the need for pandemic influenza vaccine within a timeframe that could provide significant benefit and protection for susceptible individuals and populations.
